# Supplementary material for: Mice Exposed to Combined Chronic Low-Dose Irradiation and Modeled Microgravity Develop Long-Term Neurological Sequelae
Source: Int J Mol Sci. 2019 Aug 22;20(17):4094. doi: 10.3390/ijms20174094 (PMC6747492; doi:10.3390/ijms20174094)
Supplement: Supplementary file 1 [file ijms-20-04094-s001.zip › Supplementary files/Supplemental Figure.docx]

**Supplemental Figure**

**
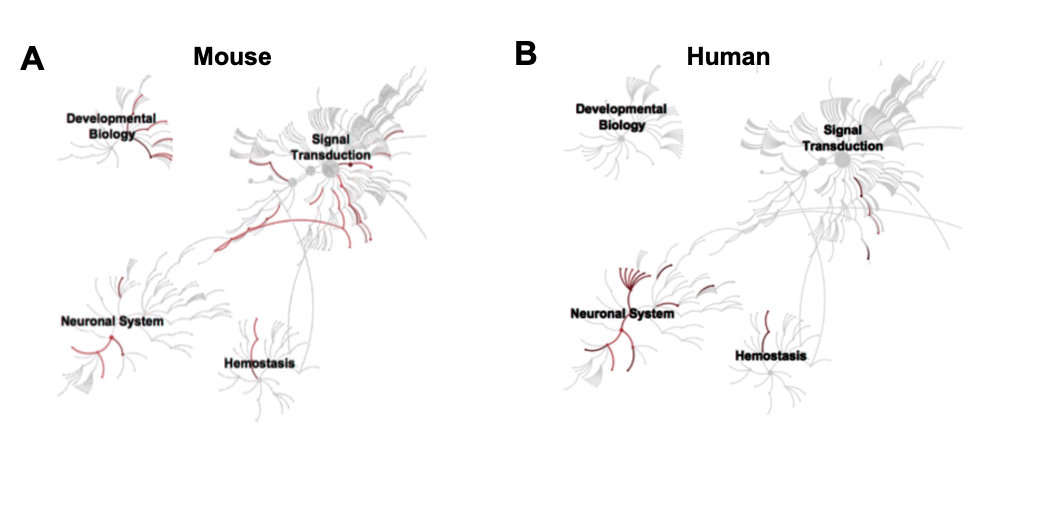
**

**Supplemental Figure 1. Abridged Reactome pathway analyses. (A)** Mouse Reactome pathway analysis for DEG. **(B)** Human Reactome pathway analysis for DEG. Reactome pathways and subpathways are displayed hierarchically. Red lines indicate aspects that are enriched in DEG; Mouse DEG were converted to their human orthologs before pathway analysis using Reactome’s “Project to human” option.
